# Autonomic Dysfunction Increases Cardiovascular Risk in the Presence of Sleep Apnea

**DOI:** 10.3389/fphys.2019.00620

**Published:** 2019-05-17

**Authors:** Javier Milagro, Margot Deviaene, Eduardo Gil, Jesús Lázaro, Bertien Buyse, Dries Testelmans, Pascal Borzée, Rik Willems, Sabine Van Huffel, Raquel Bailón, Carolina Varon

**Affiliations:** ^1^Biomedical Signal Interpretation & Computational Simulation Group, Aragón Institute of Engineering Research (I3A), IIS Aragón, University of Zaragoza, Zaragoza, Spain; ^2^Centro de Investigación Biomédica en Red en Bioingeniería, Biomateriales y Nanomedicina, Madrid, Spain; ^3^Department of Electrical Engineering-ESAT, STADIUS Center for Dynamical Systems, Signal Processing and Data Analytics, KU Leuven, Leuven, Belgium; ^4^Interuniversity Microelectronics Centre (IMEC), Leuven, Belgium; ^5^Department of Biomedical Engineering, University of Connecticut, Storrs, CT, United States; ^6^Department of Pneumology, UZ Leuven, Leuven, Belgium; ^7^Department of Cardiovascular Sciences, UZ Leuven, Leuven, Belgium

**Keywords:** heart rate variability, sleep apnea, cardiovascular disease, autonomic dysfunction, spectral analysis

## Abstract

The high prevalence of sleep apnea syndrome (SAS) and its direct relationship with an augmented risk of cardiovascular disease (CVD) have raised SAS as a primary public health problem. For this reason, extensive research aiming to understand the interaction between both conditions has been conducted. The advances in non-invasive autonomic nervous system (ANS) monitoring through heart rate variability (HRV) analysis have revealed an increased sympathetic dominance in subjects suffering from SAS when compared with controls. Similarly, HRV analysis of subjects with CVD suggests altered autonomic activity. In this work, we investigated the altered autonomic control in subjects suffering from SAS and CVD simultaneously when compared with SAS patients, as well as the possibility that ANS assessment may be useful for the early stage identification of cardiovascular risk in subjects with SAS. The analysis was performed over 199 subjects from two independent datasets during night-time, and the effects of the physiological response following an apneic episode, sleep stages, and respiration on HRV were taken into account. Results, as measured by HRV, suggest a decreased sympathetic dominance in those subjects suffering from both conditions, as well as in subjects with SAS that will develop CVDs, which was reflected in a significantly reduced sympathovagal balance (*p* < 0.05). In this way, ANS monitoring could contribute to improve screening and diagnosis, and eventually aid in the phenotyping of patients, as an altered response might have direct implications on cardiovascular health.

## 1. Introduction

Sleep apnea syndrome (SAS) is a complex sleep-related breathing disorder characterized by a repetitive total (apnea) or partial (hypopnea) upper-airway collapse (obstructive sleep apnea, OSA), an absence of respiratory drive (central sleep apnea, CSA), or a combination of both (mixed sleep apnea). During an OSA episode, forced inspiration against an obstructed upper airway leads to exaggerated negative intrathoracic pressure and is accompanied by immediate hypoxia, which triggers a complicated autonomic response (Somers et al., 2008) and large fluctuations in blood pressure (Peppard et al., 2000) and heart rate (Leung and Douglas Bradley, 2001; Caples et al., 2007). The apneic episode is often stopped by the arousal of the subject, thus resulting in a fragmented sleep. Combination of all these effects has been closely related with excessive daytime sleepiness, chronic hypertension, and increased mortality (Somers et al., 2008). Moreover, SAS has been related with a 5-fold increase in the risk for developing cardiovascular diseases (CVD), which could rise to 11-fold if not conveniently treated (Peker et al., 2002). In this way, SAS represents a well-known cause of secondary systemic and pulmonary hypertension, and a significant risk factor for coronary artery disease, cardiac arrhythmias, and heart failure (Yacoub et al., 2017; Tietjens et al., 2019). Analogously, some CVD such as heart failure, atrial fibrillation, or stroke may exert a negative effect in SAS, as a deficient blood conduction could lead to a dysregulation of PaCO_2_ and hence trigger CSA episodes (Kasai et al., 2012).

Notwithstanding the characteristic physiological response to an apneic episode shared by most of the patients, only some of them will develop CVD. Since altered heart rate variability (HRV) has been independently related to both conditions, HRV analysis has attracted widespread interest in the field of SAS (almost 200 publications in PubMed search including the key words heart rate variability and apnea, considering only the last 5 years). In this context, HRV analysis has revealed altered sympathovagal balance during sleep in subjects suffering from moderate or severe SAS when compared with healthy controls (Gula et al., 2003; Penzel et al., 2003). Also 24-h monitoring suggests altered autonomic control in SAS patients (Aydin et al., 2004), which reflects in an increased sympathetic dominance. Moreover, many physiological (e.g., hypertension, diabetes) and psychosomatic (e.g., stress, depression) conditions that constitute risk factors for CVD development, have also been related with altered HRV and sympathovagal balance (Thayer et al., 2010). Hence, HRV analysis could shed some light on the role of autonomic nervous system (ANS) in the interaction between SAS and CVD. Whereas, polysomnographic (PSG) recordings remain the gold standard for the diagnosis of SAS, it would be interesting to dispose of a simple tool for the early identification of patients at cardiovascular risk, thus improving their screening and prioritizing their treatment. If there was a relationship between ANS activity, SAS and CVD, HRV could represent such a tool. Nevertheless, previous works aiming to characterize ANS activity in SAS patients using HRV analysis usually include the apneic episodes (Gula et al., 2003; Penzel et al., 2003; Aydin et al., 2004), so that the increased sympathetic dominance observed in SAS could be biased by the sympathetic activation taking place in response to an apnea, and might not reflect the baseline state of the ANS in these subjects.

For these reasons, the aim of the present manuscript is 2-fold: first, to evaluate whether imbalanced autonomic activity could be related with CVD in SAS. Second, to investigate whether HRV analysis could be a useful tool for the early stage identification and screening of SAS patients at cardiovascular risk.

## 2. Materials and Methods

Two independent databases were employed in this study, namely the UZ Leuven and the Sleep Heart Health Study datasets. The former was employed for assessing differences in ANS activity between patients suffering from SAS or SAS plus additional cardiac comorbidities. The latter was used to see if altered ANS control can be assessed in subjects with SAS who will be latter diagnosed with a cardiovascular comorbidity. Both datasets are described below.

### 2.1. UZ Leuven Dataset

It is composed of 100 subjects (78 male, 22 female) who were referred to the sleep laboratory of the University Hospital Leuven (UZ Leuven, Leuven, Belgium) because of suspicion of SAS. PSGs were acquired, revised, and annotated by sleep specialists according to the AASM 2012 scoring rules (Berry et al., 2012). Sleep annotations included a classification of the recording period in rapid eye movement (REM) sleep and three non-REM stages (NREM1-NREM3), as well as the time occurrence and duration of each apneic/hypopneic episode and arousal. Sleep stage annotations were available for each 30-s epoch during the whole recording. In this study no difference was made between light and deep NREM sleep, so that the sleep stage classification was reduced to REM and NREM sleep. Only subjects with an apnea/hypopnea index (AHI) greater or equal than 15 were included. Bipolar ECG (lead II) and thoracic respiratory effort (recorded through respiratory inductive plethysmography) signals were acquired with a sampling frequency of 500 Hz.

The database consists of:

- 50 control patients without cardiac comorbidity (previous myocardial infarction, objective coronary disease, revascularization, or stroke) and without cardiovascular risk factors (hypertension, hyperlipidemia, diabetes), and
- 50 patients with cardiac comorbidity or cardiovascular risk factors.

Subjects in both groups were matched in age (47.8 ± 10.9 years), gender (78 males, 22 females), body mass index (BMI, 30.0 ± 4.5 kg/m^2^), and smoking habits (24 habitual smokers at the time of the recordings). The average AHI was 41.3 ± 22.0, and the average recording duration was 09:02:33 (hh:mm:ss). Demographics of each group are summarized in Table 1, where also the different medications used by the cardiac comorbidity group are indicated. Data acquisition was carried out in accordance with the recommendations of the UZ KU Leuven, Commissie Medische Ethiek. The protocol was approved by the Commissie Medische Ethiek UZ KU Leuven (ML 7962). All subjects gave written informed consent in accordance with the Declaration of Helsinki.

**Table 1:** Anthropometric data of the UZ Leuven dataset.

	**Control**	**Cardiac comorbidity**	**Total**
Number of patients	50	50	100
Age (years)	47.3 ± 10.5	48.2 ± 11.4	47.8 ± 10.9
Gender (male/female)	39 / 11	39 / 11	78 / 22
BMI (kg/m^2^)	29.9 ± 4.6	29.8 ± 4.4	30.0 ± 4.5
AHI	39.8 ± 23.3	42.7 ± 21.1	41.3 ± 22.0
Active smokers	12	12	24
Medication intake	0	33	33
• *β-blockers*	0	22	22
• *Ca channels inhibitors*	0	8	8
• *ACE inhibitors*	0	12	12
• *Diuretics*	0	4	4
• *Antidepressants*	0	1	1

*In the cardiac comorbidity group, subjects under medication intake can be treated with various distinct drugs simultaneously. BMI, Body Mass Index; AHI, Apnea Hypopnea Index; ACE, Angiotesin Converting Enzyme*.

### 2.2. Sleep Heart Health Study Dataset

The Sleep Heart Health Study (SHHS) was conducted by the National Heart Lung & Blood Institute in order to assess the negative cardiovascular effects induced by sleep-disordered breathing in general population (Quan et al., 1997). Acquisition was performed in two different sessions: a baseline session and a follow up session, performed 3–8 years after the baseline session. Despite the database is very extensive, we only considered a subset of individuals appropriate for the purpose of this study. Specifically, we were interested in those subjects who did not present any cardiac comorbidity or cardiovascular risk factor (the same ones than in the UZ Leuven dataset) at the baseline recording, but developed any of them afterwards. Conditions for inclusion were: baseline and follow up recordings available, no cardiac comorbidity or cardiovascular risk factors at baseline and subjects younger than 65 years, so that both databases were as similar as possible.

Thirty-three subjects satisfied the above mentioned criteria and suffered from a cardiac event at any point after the baseline session, so they were labeled as cardiovascular event group. Cardiac events considered for inclusion in this group were any of the following: myocardial infarction, stroke, revascularization, congestive heart failure, coronary artery disease, and procedures related with any of the previous conditions. Afterwards, one control subject without cardiac comorbidities or cardiovascular risk factors (control group) and one subject who developed cardiovascular risk factors (hypertension, hyperlipidemia, or/and diabetes) at any point after the baseline session (cardiovascular risk group) were matched to each subject in the cardiovascular event group, so that a final subset of 99 subjects was obtained. Matches were based on age (56.9 ± 4.4 years), gender (63 males, 36 females), BMI (28.1 ± 4.4 kg/m^2^), smoking habits (57 smokers at the time of the baseline session), and AHI (13.4 ± 10.9). The average recording duration was 08:27:38 (hh:mm:ss). Demographics of each group are summarized in Table 2. Since some subjects presented a low AHI, only those with AHI ≥ 5 were considered in the further analysis (AHI = 5 remains the lower limit for the diagnosis of moderate SAS). None of the subjects in the two datasets suffered from atrial fibrillation.

**Table 2:** Anthropometric data of the SHHS dataset.

	**Control**	**Cardiovascular risk**	**Cardiovascular event**	**Total**
Number of patients	33	33	33	99
Age (years)	55.8 ± 4.35	57.2 ± 4.2	57.8 ± 4.6	56.9 ± 4.4
Gender (male/female)	21 / 12	21 / 12	21 / 12	63 / 36
BMI (kg/m^2^)	28.3 ± 5.0	28.1 ± 4.5	27.9 ± 3.8	28.1 ± 4.4
AHI	13.8 ± 11.3	13.1 ± 10.1	13.3 ± 11.4	13.4 ± 10.9
Active smokers	19	19	19	57

*BMI, Body Mass Index; AHI, Apnea Hypopnea Index; ACE, Angiotesin Converting Enzyme*.

As in the UZ Leuven database, PSGs were annotated by sleep experts, and sleep stage classification (REM and NREM) was available for each 30-s interval, together with the time of occurrence and duration of each apneic/hypopneic episodes and arousal. Since SHHS has several AHI measurements available, we selected the one that best resembled the AASM 2012 scoring (containing hypopneas with arousal/desaturation >3%). Bipolar ECG (modified lead II) and thoracic respiratory effort (recorded through respiratory inductive plethysmography) were acquired at 125 and 10 Hz, respectively.

### 2.3. Preprocessing

Same preprocessing was applied to the databases described above. First, bipolar ECG signals were resampled at 1,000 Hz with cubic splines so that HRV analysis was not compromised by the effect of the sampling frequency (Merri et al., 1990). Baseline wander removal was accomplished by extracting the baseline with a low-pass filter (0.5 Hz cut-off frequency). Afterwards, the baseline was subtracted from the ECG signal.

Subsequently, QRS-complexes were detected by the wavelet-based method proposed by Martínez et al. (2004). Ectopic beat detection and correction was performed with the method described by Mateo and Laguna (2003). Essentially, it consists of thresholding instantaneous heart rate variations, so that abnormal variations are detected and labeled as ectopics. Then, ectopic beat positions and misdetections were corrected by using the heart timing signal (Mateo and Laguna, 2003).

On the other hand, respiratory effort signals were resampled at 4 Hz and respiratory rate, F_r_, was estimated from them using the method proposed by Bailón et al. (2006).

### 2.4. HRV Analysis

HRV has been largely supported as a tool for ANS assessment. In this work, the HRV representation based on the time-varying pulse integral frequency modulation (TVIPFM) model (Bailón et al., 2011) was used. Given a beat time occurrence time series ***t*** = [*t*_1_*t*_2_…*t*_*k*_…*t*_*K*_], where *k* represents the *k*-th beat and *K* is the total number of beats, the TVIPFM model allows to generalize the series as:

(1) $$\begin{array}{l} k\approx \int_{0}^{t_{k}}\frac{1+m\left(t\right)}{T\left(t\right)}dt, \end{array}$$

where the instantaneous HR is represented by the term:

(2) $$\begin{array}{l} d_{\text{HR}}\left(t\right)=\frac{1+m\left(t\right)}{T\left(t\right)}. \end{array}$$

Equation (2) is composed by two terms: the HRV signal, *m*(*t*)/*T*(*t*), and the time-varying mean HR, 1/*T*(*t*). Under the assumption that mean HR variations are slower than HRV, the latter term can be easily obtained by low-pass filtering (0.03 Hz cut-off frequency) *d*_HR_(*t*). Defining the resulting signal as *d*_HRM_(*t*) = 1/*T*(*t*), the continuous time version of the modulating signal, *m*(*t*), which contains information of ANS modulation, can be obtained as:

(3) $$\begin{array}{l} m\left(t\right)=\frac{d_{\text{HR}}\left(t\right)-d_{\text{HRM}}\left(t\right)}{d_{\text{HRM}}\left(t\right)}. \end{array}$$

Finally, *m*(*n*) was obtained by resampling *m*(*t*) at 4 Hz.

Each 60 s HRV power spectral density, Ŝ_HRV_(*j*, F), was estimated from the *j*-th segment of length 5 min of *m*(*n*) by the Welch's periodogram. Fifty-second Hamming windows with 50% overlap were employed. Subsequently, the spectral indexes were computed from Ŝ_HRV_(*j*, F).

Low-frequency (LF) power, P_LF_, was defined as the power in the classical LF band ([0.04, 0.15] Hz) (Task Force, 1996). A preliminary analysis of the respiratory rate revealed some values close to 0.4 Hz, which remains the upper limit of the classical high-frequency (HF) band ([0.15, 0.4] Hz) and could lead to an underestimation of the HF power (P_HF_) (Bailón et al., 2007). For this reason, two different alternative definitions of the HF band were used:

(4) $$\begin{array}{l} \Omega_{\text{HF}}^{c}\left(j\right)=\left[\max\left(0.15,{\bar{\text{F}}}_{\text{r}}\left(j\right)-0.125\right),{\bar{\text{F}}}_{\text{r}}\left(j\right)+0.125\right]\text{Hz}\\ \Omega_{\text{HF}}^{e}\left(j\right)=\left[0.15,\bar{\text{HR}}\left(j\right)/2\right]\text{Hz}, \end{array}$$

where $\Omega_{\text{HF}}^{c}$ and $\Omega_{\text{HF}}^{e}$ stand for centered in F_r_ and extended HF band respectively, and ${\bar{\text{F}}}_{\text{r}}\left(j\right)$ and $\bar{\text{HR}}\left(j\right)$ account for the mean respiratory rate and HR in the *j*-th 5-min window. ${\text{P}}_{\text{HF}}^{c}$ and ${\text{P}}_{\text{HF}}^{e}$ were defined as the power within $\Omega_{\text{HF}}^{c}$ and $\Omega_{\text{HF}}^{e}$, respectively. In addition, LF to HF ratio, R_LF/HF_ = P_LF_/P_HF_, and normalized LF power, P_LFn_ = P_LF_/(P_LF_+P_HF_) were also redefined to account for the different versions of the HF band, so that ${\text{R}}_{\text{LF/HF}}^{c}$ and ${\text{P}}_{\text{LFn}}^{c}$ refer to $\Omega_{\text{HF}}^{c}$ whereas ${\text{R}}_{\text{LF/HF}}^{e}$ and ${\text{P}}_{\text{LFn}}^{e}$ are associated with $\Omega_{\text{HF}}^{e}$.

Also very low-frequency (VLF) power (P_VLF_) was considered, being it defined as the power of *d*_HRM_, in order to account for the slower variations of *m*(*n*). Finally, mean normal-to-normal interval (NN) was defined as the mean RR interval within each 5-min window (Task Force, 1996).

### 2.5. Effect of Sleep Stages on HRV

Sleep stages are known to exert an important effect on HRV, which is mainly reflected as an increased parasympathetic activity during NREM sleep and an awake-like sympathetic activity during REM sleep (Somers et al., 1993; Bušek et al., 2005). These large inter-stage fluctuations make it advisable to consider sleep stages in the analysis. In this way, HRV analysis was performed for NREM and REM sleep separately, by considering PSG-based sleep stage scoring.

### 2.6. Effect of Apneas, Hypopneas, and Arousals on HRV

The complex physiological response to an apnea or hypopnea usually finishes with an increase in sympathetic activity that may trigger an arousal, thus biasing any possible measurement in that period toward high sympathetic activity. Despite this well-known effect, apneic episodes are usually included in the analysis. A major innovation of this work is that the episodes of apneas, hypopneas, and arousals (for simplicity summarized as apneic episodes hereon) were removed from the analysis, so that ANS activity can be assessed in a more basal state.

In order to minimize the effects of the recovery after an apneic episode, the 1 min after the offset of each event was also removed, since the tachycardia following an apnea or arousal often lasts about 20–30 s (Stein and Pu, 2012). Some subjects presented an extremely high number of events, and hence only a few 5-min apneic episodes-free segments were usable (especially during REM sleep, which is a shorter stage and with higher incidence of apneic episodes). Thus, and to guarantee a minimum sample size, subjects with less than 10 five-minute segments were discarded.

**Figure 1:**
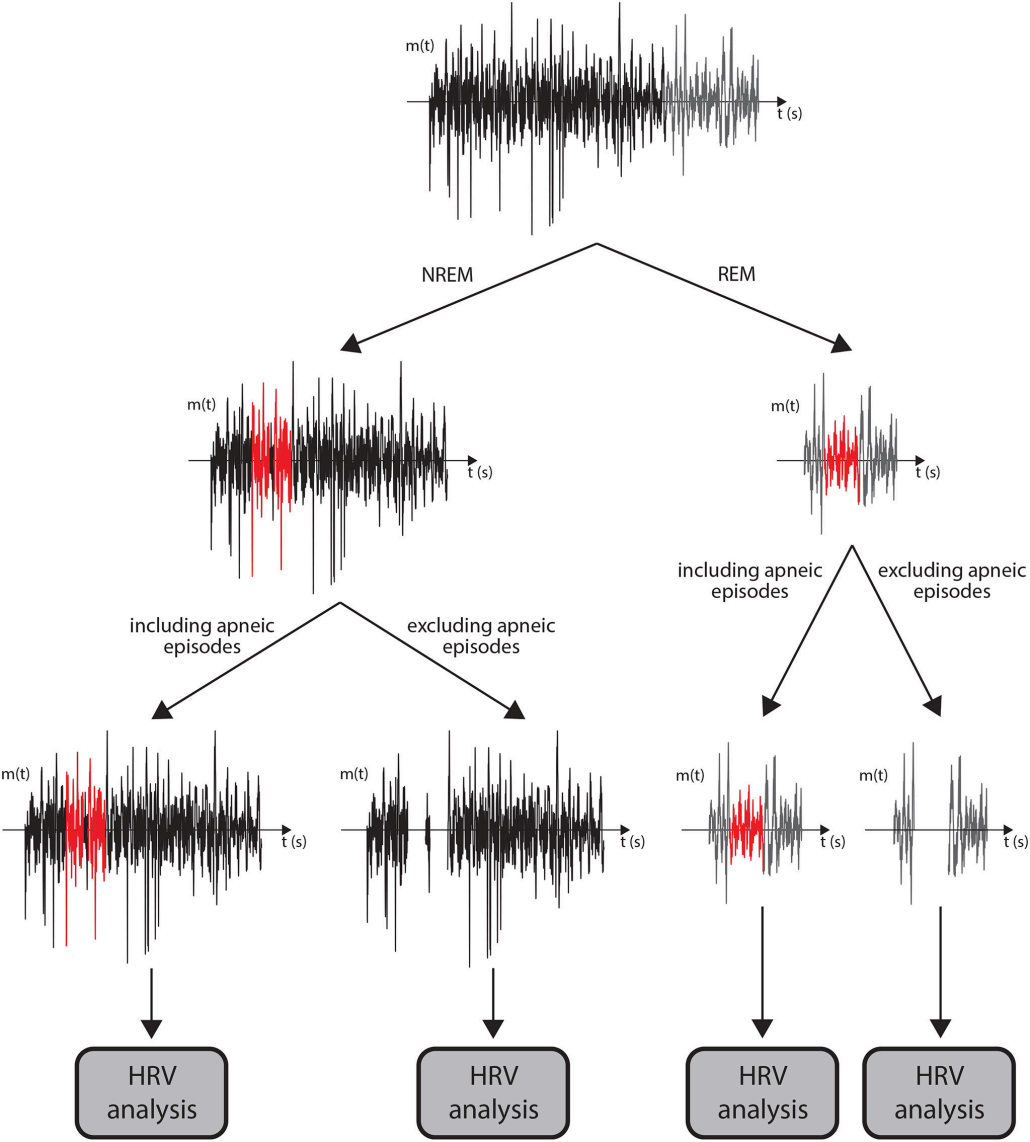
A flowchart of the data analysis performed for each subject is displayed. First, the modulating signal was divided in periods corresponding to NREM (black) and REM (gray) sleep. Afterwards, two different HRV analyses were performed in each of the sleep stages: one including the apneic episodes (red) and one excluding them (the 1-min period after the apneic episodes are included in the segments highlighted in red). The HRV analyses are performed over 5-min windows of available signal.

Nevertheless, the analysis was repeated including the apneic episodes, so that the results were comparable with previous studies. A schematic of the different proposed analyses is depicted in Figure 1.

### 2.7. Effect of Medication

Patients in the cardiac comorbidity group of the UZ Leuven dataset suffering from hypertension (33 out of 50) were under anti-hypertensive medication at the time of the study. Each patient was administered a different drug or combination of drugs such as β-blockers, calcium channels inhibitors or blockers, angiotensin converting enzyme inhibitors, and diuretics, which are summarized in Table 1. Since anti-hypertensives could directly alter HRV measurements (Guzzetti et al., 1988; Bekheit et al., 1990), we considered medication intake as a possible confounder in the analysis.

The effect of medication was analyzed in the following manner. First, patients with cardiac comorbidities were divided in two subgroups: under and not under anti-hypertensive drugs intake. Afterwards, the differences of the mean NN and ${\text{P}}_{\text{LFn}}^{e}$ between each subject and his/her matched control were computed, and the distributions obtained for the two subgroups were compared.

### 2.8. Statistical Methods

The mean value of each parameter for the different sleep stages was obtained for each subject. Normality of the data was rejected using a Kolmogorov-Smirnov test (*p* < 0.05) and so a paired Wilcoxon signed-rank test was applied in order to assess differences between the matched groups. This test was applied twice: once considering apneas, hypopneas, and arousals, and another time excluding them from the analysis. When the comparison was between not matched groups, a two-sided Wilcoxon rank-sum test was applied instead. Significance level for considering statistical differences between groups was set to 0.05.

## 3. Results

In both datasets, results of the HRV analysis were similar when defining the HF band as $\Omega_{\text{HF}}^{e}$ or $\Omega_{\text{HF}}^{c}$ so, for simplicity, only those concerning the former are presented. The results obtained for each of the two datasets are summarized below.

### 3.1. UZ Leuven Dataset

**Figure 2:**
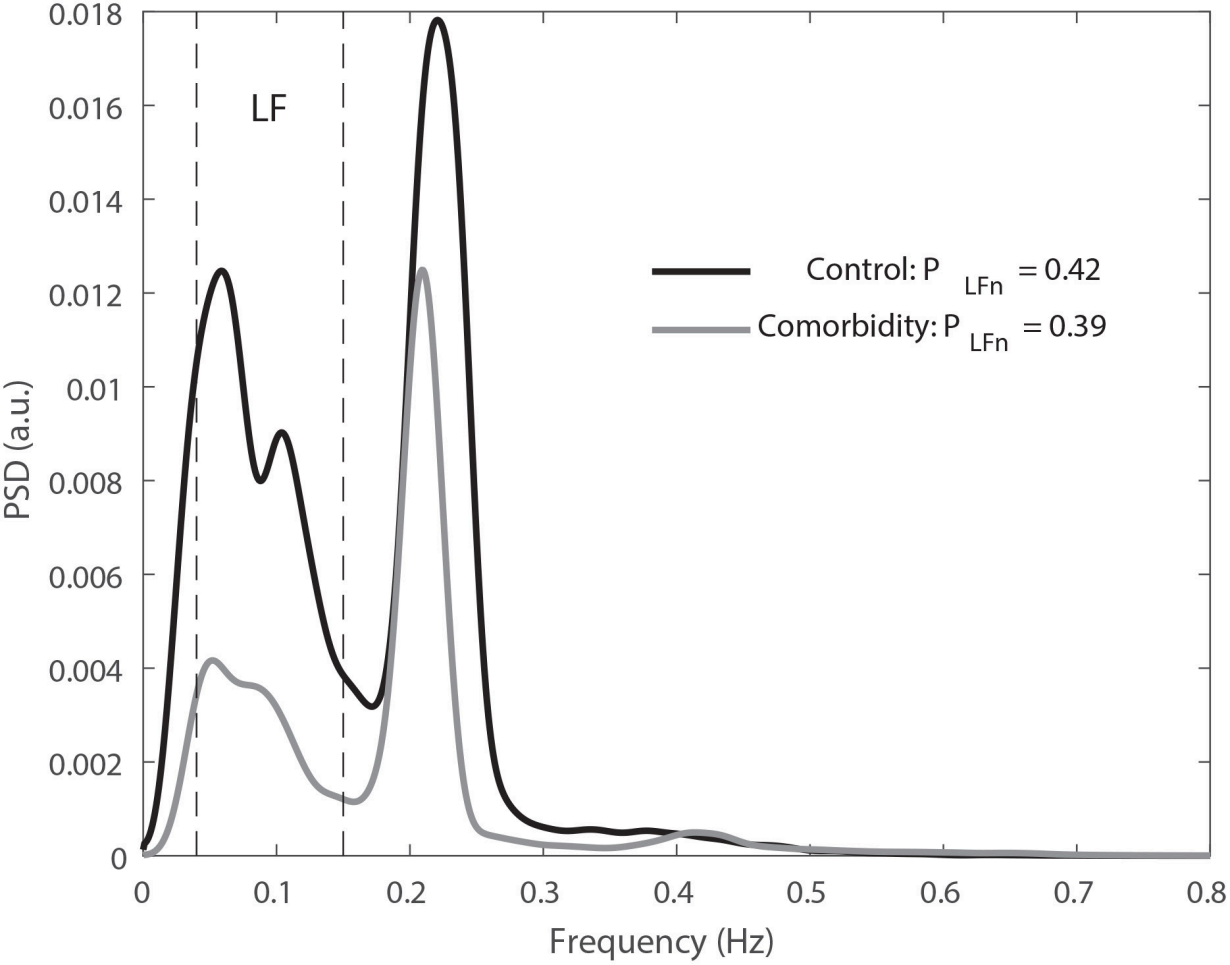
Average spectra for all the NREM segments (excluding those with apneic episodes) of a control subject of the UZ Leuven dataset (black) and his match (gray) are displayed. An increased sympathetic dominance can be noticed in the control subject, as reflected by the relative higher low frequency power content. The dashed black lines indicate the boundaries of the low frequency band. The number of averaged 5 min segments was 50 and 36 for the control and the match, respectively. As estimated from the modulating signal, the power spectral density is given in arbitrary units (a.u.).

The results of the HRV analysis including and excluding apneic episodes are presented in Table 3. A tendency toward lower values of ${\text{R}}_{\text{LF/HF}}^{e}$ and ${\text{P}}_{\text{LFn}}^{e}$ in the cardiac comorbidity group than in the control group was assessed when excluding apneic episodes from the analysis. These differences were statistically significant during NREM sleep. An example of the mean overnight spectra of a control subject and his/her comorbidity match during NREM sleep is displayed in Figure 2. Similar results were obtained when including apneic episodes, although significant differences were only assessed during REM sleep in this case. Regarding the differences between sleep stages, decreased NN and ${\text{P}}_{\text{HF}}^{e}$ and increased ${\text{R}}_{\text{LF/HF}}^{e}$ and ${\text{P}}_{\text{LFn}}^{e}$ were assessed during REM sleep. When excluding apneic episodes from the analysis, also F_r_ was increased during REM sleep.

**Table 3:** Results of HRV analysis for the UZ Leuven dataset.

	**Control**		**Cardiac comorbidity**	
	**NREM**	**REM**	**NREM**	**REM**
**EXCLUDING APNEIC EPISODES**				
F_r_ (Hz)	0.23 (0.06)	0.25 (0.06)^*^	0.23 (0.07)	0.25 (0.08)^*^
NN (ms)	920.13 (184.89)	939.09 (178.61)^*^	951.08 (173.29)	911.34 (177.57)^*^
P_VLF_ (a.u.)	1.16 (0.40)	1.12 (0.40)^*^	1.09 (0.42)	1.20 (0.41)^*^
P_LF_ (a.u.)	0.0005 (0.0006)	0.0006 (0.0015)	0.0005 (0.0007)	0.0008 (0.0013)
${\text{P}}_{\text{HF}}^{e}$ (a.u.)	0.0005 (0.0010)	0.0003 (0.0005)^*^	0.0006 (0.0016)	0.0005 (0.0012)^*^
${\text{R}}_{\text{LF/HF}}^{e}$ (n.u.)	0.96 (1.12)	2.43 (3.14)^*^	0.69 (0.94)^†^	1.52 (1.77)^*^
${\text{P}}_{\text{LFn}}^{e}$ (n.u.)	0.47 (0.24)	0.68 (0.22)^*^	0.38 (0.23)^†^	0.60 (0.19)^*^
N (subjects)	43	29	42	25
**INCLUDING APNEIC EPISODES**				
F_r_ (Hz)	0.23 (0.05)	0.23 (0.07)	0.23 (0.06)	0.24 (0.07)
NN (ms)	938.53 (200.44)	936.66 (198.97)^*^	950.39 (155.86)	900.00 (125.60)^*^
P_VLF_ (a.u.)	1.13 (0.42)	1.12 (0.48)^*^	1.10 (0.37)	1.23 (0.32))^*^
P_LF_ (a.u.)	0.0011 (0.0013)	0.0012 (0.0020)	0.0010 (0.0015)	0.0009 (0.0014)^†^
${\text{P}}_{\text{HF}}^{e}$ (a.u.)	0.0006 (0.0012)	0.0004 (0.0006)^*^	0.0008 (0.0016)	0.0005 (0.0009)^*^
${\text{R}}_{\text{LF/HF}}^{e}$ (n.u.)	1.79 (2.56)	3.16 (3.41)^*^	1.42 (1.50)	1.72 (2.32)^†^, ^*^
${\text{P}}_{\text{LFn}}^{e}$ (n.u.)	0.59 (0.28)	0.72 (0.20)^*^	0.52 (0.21)	0.60 (0.22)^†^, ^*^
N (subjects)	46	50	49	46

Results are displayed as median (interquartile range), except for the number of subjects. Significant differences with the same sleep stage of the control group are marked with ^†^ (p < 0.05). Significant differences between NREM and REM sleep within each group are marked with ^*^ (p < 0.05).

**Figure 3:**
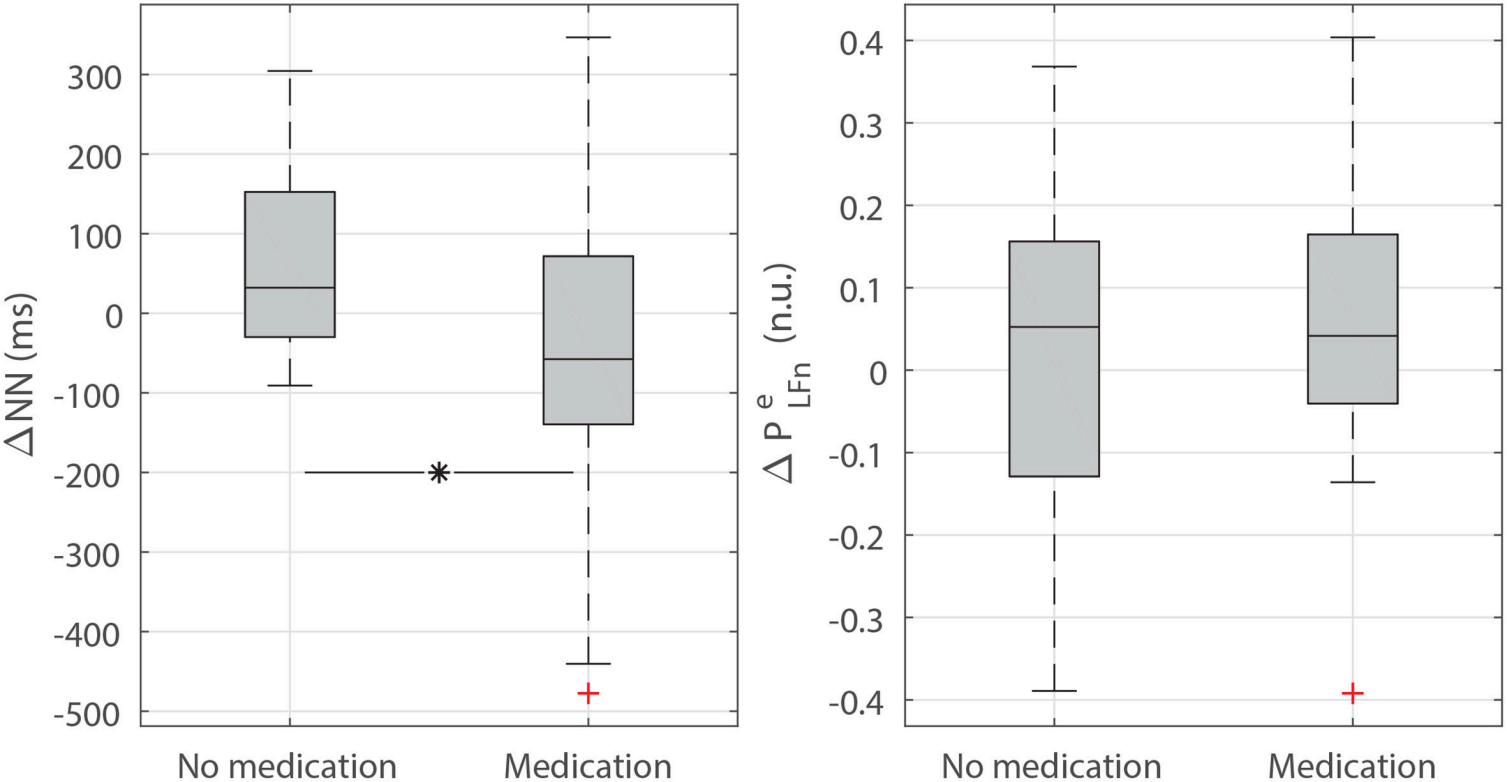
Boxplots of the differences in mean NN (ΔNN) and ${\text{P}}_{\text{LFn}}^{e}$ (Δ${\text{P}}_{\text{LFn}}^{e}$) between the control subjects of the UZ Leuven dataset and their matches under or not under medication intake (during NREM sleep and excluding apneic episodes). Whereas, ΔNN is increased in the group under medication intake when compared to the group without medication (*p* < 0.05, indicated with ^*^), no differences in Δ${\text{P}}_{\text{LFn}}^{e}$ were assessed.

The results obtained for the subgroups under and not under medication intake are displayed in Figure 3. Whereas a higher NN was assessed in the subgroup with medication, no differences were found regarding ${\text{P}}_{\text{LFn}}^{e}$ (although in Figure 3 only the analysis during NREM sleep and excluding apneic episodes is represented, no differences in ${\text{P}}_{\text{LFn}}^{e}$ were found for REM sleep nor when including apneic episodes).

### 3.2. SHHS Dataset

The results of the HRV analysis are summarized in Table 4. An increased ${\text{P}}_{\text{HF}}^{e}$ and decreased ${\text{R}}_{\text{LF/HF}}^{e}$ and ${\text{P}}_{\text{LFn}}^{e}$ were observed in the cardiovascular risk and cardiovascular event groups when compared to controls when excluding the apneic episodes. In the cardiovascular event group, those differences turned statistically significant for ${\text{P}}_{\text{LFn}}^{e}$ during NREM sleep. Similar results were obtained when including apneic episodes in the analysis. In general, differences between sleep stages were noticed as decreased NN and ${\text{P}}_{\text{HF}}^{e}$ and increased ${\text{P}}_{\text{LF/HF}}^{e}$ and ${\text{R}}_{\text{LFn}}^{e}$ during REM sleep. However, higher NN and lower P_VLF_ were assessed during REM than during NREM sleep in some cases (Tables 3, 4), but this is most likely due to the reduced number of segments at REM sleep available for the analysis.

**Table 4:** Results of HRV analysis for the SHHS dataset.

	**Control**		**Cardiovascular risk**		**Cardiovascular event**	
	**NREM**	**REM**	**NREM**	**REM**	**NREM**	**REM**
**EXCLUDING APNEIC EPISODES**						
F_r_ (Hz)	0.25 (0.05)	0.24 (0.03)	0.24 (0.04)	0.25 (0.03)	0.25 (0.05)	0.24 (0.07)
NN (ms)	925.95 (150.24)	924.58 (123.65)	980.00 (168.87)	959.41 (266.71)	914.59 (201.34)	919.62 (186.39)^*^
P_VLF_ (a.u.)	1.16 (0.35)	1.15 (0.33)	1.03 (0.34)	1.07 (0.60)	1.18 (0.47)	1.17 (0.51)^*^
P_LF_ (a.u.)	0.0004 (0.0004)	0.0005 (0.0009)	0.0004 (0.0005)	0.0004 (0.0026)	0.0003 (0.0002)	0.0003 (0.0003)
${\text{P}}_{\text{HF}}^{e}$ (a.u.)	0.0002 (0.0003)	0.0001 (0.0002)	0.0004 (0.0005)	0.0006 (0.0007)	0.0003 (0.0005)	0.0001 (0.0001)^*^
${\text{R}}_{\text{LF/HF}}^{e}$ (n.u.)	1.13 (1.01)	3.99 (2.62)^*^	0.94 (1.21)	2.01 (2.38)	0.97 (0.99)	1.65 (4.06)
${\text{P}}_{\text{LFn}}^{e}$ (n.u.)	0.49 (0.17)	0.79 (0.11)^*^	0.45 (0.28)	0.67 (0.30)^*^	0.43 (0.28)^†^	0.59 (0.36)
N (subjects)	23	6	24	7	22	6
**INCLUDING APNEIC EPISODES**						
F_r_ (Hz)	0.26 (0.05)	0.25 (0.04)	0.24 (0.04)	0.26 (0.04)	0.25 (0.05)	0.25 (0.04)
NN (ms)	922.36 (158.63)	929.32 (154.77)	992.65 (144.38)	1016.15 (134.91)^*^	929.90 (217.02)	897.97 (154.08)
P_VLF_ (a.u.)	1.16 (0.39)	1.15 (0.36)	1.01 (0.30)	0.96 (0.26)^*^	1.16 (0.52)	1.22 (0.42)
P_LF_ (a.u.)	0.0005 (0.0006)	0.0005 (0.0006)	0.0006 (0.0008)	0.0007 (0.001)	0.0004 (0.0005)	0.0005 (0.0007)
${\text{P}}_{\text{HF}}^{e}$ (a.u.)	0.0002 (0.0003)	0.0002 (0.0001)^*^	0.0005 (0.0005)	0.0004 (0.0004)^†^	0.0004 (0.0005)	0.0002 (0.0001)^*^
${\text{R}}_{\text{LF/HF}}^{e}$ (n.u.)	1.74 (1.08)	3.19 (2.94)^*^	1.39 (1.26)	2.09 (2.22)^*^	1.09 (1.27)^†^	2.86 (2.25)^*^
${\text{P}}_{\text{LFn}}^{e}$ (n.u.)	0.55 (0.13)	0.74 (0.18)^*^	0.53 (0.24)	0.66 (0.22)^*^	0.46 (0.24)^†^	0.67 (0.26)^*^
N (subjects)	25	23	26	25	25	22

Results are displayed as median (interquartile range), except for the number of subjects. Significant differences with the same sleep stage of the control group are marked with ^†^ (p < 0.05). Significant differences between NREM and REM sleep within each group are marked with ^*^ (p < 0.05).

## 4. Discussion

The main purpose of the present study was to assess whether imbalanced autonomic activity could be related to CVD in patients with SAS, as well as to investigate the potential use of ANS activity analysis in the early stage identification of patients at higher cardiovascular risk. ANS evaluation was achieved by HRV analysis, since it has been largely supported as a non-invasive tool for ANS activity assessment (Task Force, 1996). However, HRV should be addressed carefully in nocturnal recordings, since several studies have reported differences in HRV among the different sleep stages (Somers et al., 1993; Bušek et al., 2005). Also differences in HRV when comparing subjects with and without apneas have been described in the literature (Gula et al., 2003; Penzel et al., 2003). Nevertheless, whereas the effect of sleep stages is often considered in overnight HRV analysis, the effect of apneic episodes has been largely ignored. In this way, increased sympathetic dominance assessed in SAS patients might be reflecting the adrenergic surge following apneas and not a chronic sympathetic nervous system (SNS) dominance during rest. For this reason, in this work we proposed to discard apneic episodes from the analysis, so that ANS evaluation is performed during the most basal condition.

The effect of excluding apneic episodes from the analysis can be noticed in both datasets (Tables 3, 4), with large significant differences in P_LF_ and sympathovagal balance measurements. The apparent reduction observed in sympathetic activity when not considering the periods of apnea suggests that apneic episodes do alter ANS assessment by HRV and hence should be removed from the analysis, since changes in sympathetic activity unrelated to apneas might be masked otherwise. Moreover, this effect was more evident for the patients in the UZ Leuven dataset, with larger AHI than in the SHHS.

With respect to the different definitions of the HF band employed in HRV analysis, $\Omega_{\text{HF}}^{e}$ resulted to be the most discriminative between groups, specially when apneic episodes were excluded from the analysis (although similar results were achieved with $\Omega_{\text{HF}}^{c}$). The motivation for considering modified HF bands was that a preliminary analysis of F_r_ revealed the existence of some high values, which could yield to a power shift outside the band and hence result in underestimations of the real power. In this way, one possible solution is to center the band into the estimated respiratory rate, so that respiratory-related power lays inside the band. Besides, the fact that better results were obtained when considering $\Omega_{\text{HF}}^{e}$ could be related with differences in HR (as the higher limit of the extended band was selected as $\bar{\text{HR}}/2$ Hz, since HR remains the intrinsic sampling rate of HRV; Laguna et al., 1998), although the absence of significant differences in the NN of the distinct groups in both datasets suggests that this is not a likely explanation for the obtained results. Alternatively, the non-linear interaction between HR and respiration during sleep (Varon and Van Huffel, 2017) may result in important frequency components that lie outside the classic and the centered bands.

### 4.1. UZ Leuven Dataset

In order to study the relationship between ANS, SAS, and CVD, we considered the UZ Leuven database described in section 2.1, since it is composed by SAS patients with and without cardiac comorbidities that were matched based on age, gender, BMI, and smoking habits. When comparing the patients with cardiac comorbidities with their matched controls a decreased sympathovagal balance, as assessed by lower values of ${\text{R}}_{\text{LF/HF}}^{e}$ and ${\text{P}}_{\text{LFn}}^{e}$, was observed in the former (Table 3). This decreased sympathetic dominance, exemplified in Figure 2, could reflect a lack of adaptability of ANS and hence incapability to restore homeostasis after an apneic episode. If this was the case, an inefficient response to oxygen deprivation could directly affect the cardiovascular system, leading to inflammation (Somers et al., 2008), oxidative stress (Suzuki et al., 2006), or tonic chemoreceptor activation (Narkiewicz et al., 1998) among others, which are intrinsically related with the development of CVDs (Somers et al., 2008). The fact that statistically significant differences were observed in NREM when excluding apneic episodes from the analysis but not when including them might suggest that the sympathetic activations following apneas could be masking the lowered sympathetic dominance in the comorbidity group. On the other hand, the increased incidence of apneic episodes during REM sleep (Sackner et al., 1975) results in a reduction in the number of subjects considered in the analysis when excluding them, which could explain the absence of significant differences during this sleep stage. Similarly to previous studies (Somers et al., 1993; Bušek et al., 2005), a higher sympathetic tone was assessed during REM than during NREM sleep, as reflected in increased P_LF_, ${\text{R}}_{\text{LF/HF}}^{e}$, and ${\text{P}}_{\text{LFn}}^{e}$ and decreased NN and ${\text{P}}_{\text{HF}}^{e}$ in the former.

Nevertheless, altered sympathovagal balance should be regarded carefully, as 33 out of 50 patients in the cardiac comorbidity group were under anti-hypertensive medication at the time of the recordings. Since anti-hypertensives could contribute to reduced cardiac sympathetic activity (Guzzetti et al., 1988; Bekheit et al., 1990), a more detailed analysis was performed in order to check whether the observed differences could be explained by medication intake. In this way, the differences in mean NN and ${\text{P}}_{\text{LFn}}^{e}$ of the patients under medication and their matched controls were compared to those of the patients without medication (Figure 3). The results revealed a higher NN, i.e., a lower HR, in those patients under anti-hypertensives, as expected, although no differences were found in ${\text{P}}_{\text{LFn}}^{e}$. The lowered NN in the medication group would be reflected as an increase in the mean HR which is corrected in the TVIPFM model (see Equation 3) and hence is not expected to have a big influence in the analysis. On the other hand, ${\text{P}}_{\text{LFn}}^{e}$ was apparently independent of the use of medication, possibly due to the mentioned correction by mean HR intrinsic to the TVIPFM model.

### 4.2. SHHS Dataset

Moreover, in order to evaluate if altered ANS activity may be prior to cardiovascular disorders in subjects with SAS, a second dataset consisting of a subset of the SHHS and described in section 2.2 was considered. Again, HRV analysis revealed a decreased sympathetic dominance in the cardiovascular risk and cardiovascular event groups (Table 4), which turned statistically significant in the case of the cardiovascular event group (during NREM sleep). Given that subjects in the cardiovascular event group presented an altered sympathovagal balance when compared with their matched controls, despite the fact that they did not suffer from any CVD at the time of the recording, it is possible that individuals with SAS and altered sympathovagal balance are at augmented risk for developing CVDs. This unbalanced sympathovagal activity may be an indicator of either a lowered SNS activity, a dysfunction in the response to SNS stimuli or a combination of both. Although decreased LF variability has been assessed in severe chronic heart failure (Van De Borne et al., 1997), this effect appears to be visible only in the most advanced stages of the disease. Nonetheless, the desensitization of β-adrenergic receptors when subjected to a recurrent stimuli (Barnes, 1995) could point to SAS as a possible precursor of CVD, as heart damage has been associated with decreased β-adrenergic receptors density and decreased sensitivity to adrenergic stimulation (Bristow et al., 1982). Regarding the differences between sleep stages, increased sympathetic dominance was generally observed during REM sleep as expected.

### 4.3. Limitations

There are some limitations in this study that must be mentioned. The first and most important one is the use of anti-hypertensive medication by a large subset of subjects in the UZ Leuven database, which might compromise the physiological interpretation. Although differences that may be induced by medication intake were analyzed carefully, it is not possible to ensure that it does not have an effect on the results. Moreover, there is controversy in the literature, with some studies reporting absence of changes in the sympathovagal balance in subjects under β-blockers (Goldsmith et al., 1997; Malfatto et al., 1998), and some others suggesting altered sympathetic dominance (Sandrone et al., 1994; Lin et al., 1999). Another limitation is that the proposed analysis for ANS assessment is only valid during sinus rhythm and it is not applicable to other scenarios. This limitation takes special relevance in the case of atrial fibrillation, since it is known to be associated with SAS (Tietjens et al., 2019). Regarding sleep stages, no distinction was made between light and deep NREM sleep due to the extremely low number of deep sleep epochs (less than 5% of the recording duration in most of the subjects in the UZ Leuven dataset, prior to apneic epochs deletion). In the SHHS dataset, the low number of analyzed subjects during REM sleep after removing apneic episodes compromises the further physiological interpretation. On the other hand, whereas the results obtained for both datasets are coherent, the datasets are not comparable, due to differences in mean age and AHI, and to the fact that cardiac comorbidity subjects in the UZ Leuven dataset had already developed CVDs. It is also important to highlight that subjects in UZ Leuven attended to the sleep laboratory because of complains and/or symptoms related to SAS, whereas volunteers in SHHS did not report any interference with their daily life, regardless of their scored AHI. Finally, and although several cardiac conditions with different origin and effects were considered simultaneously, the scope of this work was limited to the risk of developing CVDs as a whole.

## 5. Conclusion

The combination of all the underlying mechanisms that act in response to an apneic episode, together with the functional alterations caused by the different CVD, result in a very complex frame that obscures the physiological interpretation. Despite, decreased sympathetic dominance was assessed in SAS patients suffering from cardiac comorbidities. Furthermore, retrospective analysis of the subjects with SAS that will develop cardiovascular events in the future also revealed a reduced sympathetic dominance. Notwithstanding that further work is needed in the field of SAS phenotyping, HRV analysis could represent a useful tool for improving the screening and diagnosis of SAS patients with increased cardiovascular risk. Moreover, the importance of considering the effect of the apneic episodes in the interpretation of HRV analysis was addressed.

## Ethics Statement

This study was carried out in accordance with the recommendations of the UZ KU Leuven, Commissie Medische Ethiek. The protocol was approved by the Commissie Medische Ethiek UZ KU Leuven (ML 7962). All subjects gave written informed consent in accordance with the Declaration of Helsinki.

## Author Contributions

All authors equally contributed to the conception of the work, revising it critically for important intellectual content, final approval of the version to be published, and to the discussion and interpretation of the results. Additionally, EG, SV, RB, and CV supervised this work, also giving methodological support. BB, DT, and PB were responsible of the UZ Leuven data acquisition, also contributing with clinical support. MD prepared the datasets for the analysis. JL and RW contributed with methodological and clinical support. Finally, JM was responsible for drafting this work.

### Conflict of Interest Statement

The authors declare that the research was conducted in the absence of any commercial or financial relationships that could be construed as a potential conflict of interest.

## Abbreviations

AHI: Apnea Hypopnea Index
ANS: Autonomic Nervous System
BMI: Body Mass Index
CSA: Central Sleep Apnea
CVD: Cardiovascular Disease
ECG: Electrocardiogram
HF: High Frequency
HR: Heart Rate
HRV: Heart Rate Variability
LF: Low Frequency
NREM: Non-Rapid Eye Movement
OSA: Obstructive Sleep Apnea
PSG: Polysomnography
REM: Rapid Eye Movement
SAS: Sleep Apnea Syndrome
SHHS: Sleep Heart Health Study
SNS: Sympathetic Nervous System
TVIPFM: Time Varying Integral Pulse Frequency Modulation
VLF: Very Low Frequency.
